# Cytoplasmic fluidity couples nutrient availability and enterocyte fate in vivo

**DOI:** 10.1073/pnas.2602724123

**Published:** 2026-08-04

**Authors:** Yukana Nakamura, Motohiro Morikawa, Tomomi Takano, Sa Kan Yoo

**Affiliations:** ^a^ Graduate School of Science and Technology, Kwansei Gakuin University, Sanda 669-1330, Japan; ^b^ Laboratory for Homeodynamics, RIKEN Center for Biosystems Dynamics Research, Kobe 650-0047, Japan; ^c^ Division of Developmental Biology and Regenerative Medicine, Kobe University, Kobe 650-0047, Japan

**Keywords:** nutrient, *Drosophila*, gut, enterocyte, viscosity

## Abstract

Cells must continuously adapt their internal state to fluctuating nutritional environments. For the adaptation, cells sense distinct nutrients through specific molecular signals, such as the insulin and mTOR pathways. Here, in addition to the conventional nutrient-sensing mechanisms, we reveal a mechanistic layer by which gut enterocytes respond to dietary contents, demonstrating that nutritional components in food regulate cytoplasmic fluidity, a fundamental biophysical property and determine cellular status. We found that the quantity, rather than the quality, of amino acids alters the nanoscale cytoplasmic fluidity in *Drosophila* enterocytes and the frequency of erebosis, a nonapoptotic cell death mediating intestinal cell turnover. Manipulating cytoplasmic fluidity through several independent inert small viscogen molecules affects erebosis, indicating that intracellular fluidity can directly control cell fate decisions. We propose that intracellular nanoscale fluidity represents a fundamental principle for enterocytes to detect and adapt to dietary components, providing a biophysical basis for cellular homeostasis in vivo.

## Significance

We demonstrate that fly gut cells possess a system to detect the quantity rather than the quality of molecules in food through changes in cytoplasmic fluidity and alter their cell fate accordingly. This provides an additional mechanistic layer of cellular responses to dietary components, beyond conventional nutrient-sensing pathways activated by specific nutrients. Our study suggests that cytoplasmic fluidity can serve as an integrative biophysical readout of the dietary environment.

In response to varying external nutrient conditions, cells must constantly adjust their internal state. In general, when cells require more energy during starvation or when nutrients are abundant and additional uptake is unnecessary under satiety, their internal states differ accordingly (1).

Gut epithelial cells constitute the first line of cells directly exposed to external nutrients and must continuously adapt their status according to both extracellular and intracellular nutritional conditions. Intestinal cells have one of the shortest lifespans and are thus in a constant state of flux (2). We recently found that *Drosophila* gut enterocyte turnover is mediated by a nonapoptotic, non-necrotic, and nonautophagic process termed erebosis (3). Since the status of gut cells can be generally regulated by external nutritional conditions (4), we became interested in whether external nutrients in the food affect erebosis.

Traditionally, cellular responses to different types of nutrients have been understood primarily through molecular signaling pathways, such as insulin and TOR pathways, which are elicited by specific nutrients (5). Glucose is sensed by ChREBP/MondoA and also stimulates insulin secretion (6), whereas amino acids activate specific sensors that contribute to TOR activation (7). These signaling pathways are involved in nutrient-dependent cell fate decision and the gut growth (4, 8, 9). We investigated how dietary nutrients regulate gut cell status by examining effects of nutrients on erebosis.

## Results

**Fig. 1.**
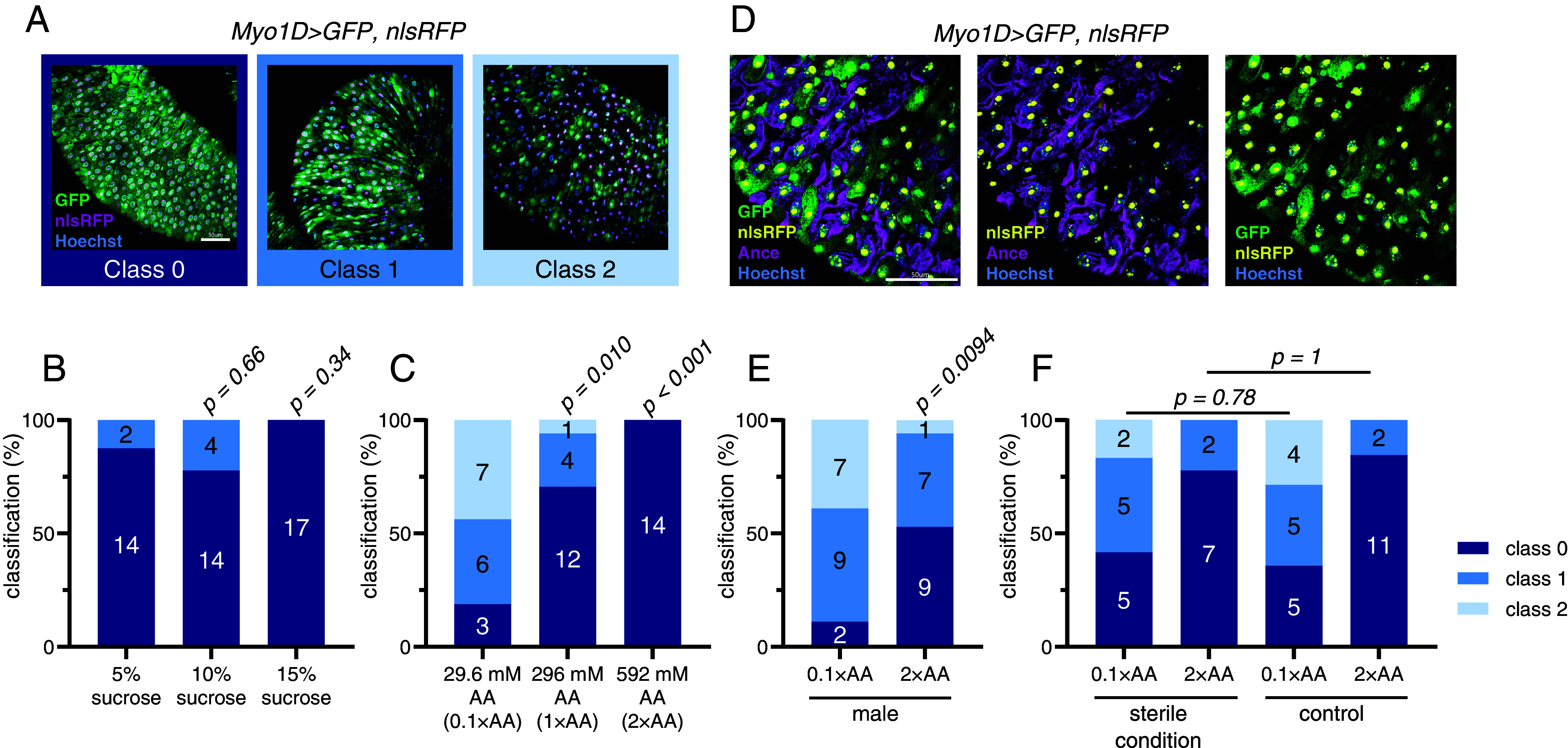
Low amino acid food induces erebosis. (*A*) Classification of erebosis based on the number of GFP- nlsRFP+cells: class0- three or fewer erebotic cells/view, class1- intermediate between class0 and 2, class2- >30% erebotic cells/view. (*B*) Altering sucrose concentrations in the food does not affect erebosis. (*C*) Altering amino acid concentrations in the food affects erebosis. (*D*) GFP- cells under the low amino acid condition have Ance, an erebosis marker, indicating that they are erebotic cells. (*E*) Altering amino acid concentrations in the food affects erebosis in male guts. (*F*) Altering amino acid concentrations in the food under the sterile condition has similar effects on erebosis as under conventional conditions. (Scale bars, 50 μm.) *P* values were calculated using Fisher’s exact test and adjusted with the Benjamini–Hochberg method for multiple comparisons.

We examined whether changes in nutrient composition, while maintaining the same caloric content, affect erebosis. To analyze erebosis, we used a transgenic *Drosophila* stock expressing GFP and nuclear RFP (nlsRFP) in enterocytes under the control of *Myo1D-Gal4*, as previously described (3). We found that a low-yeast, high-sugar diet induced erebosis even when the total caloric content was constant (*SI Appendix*, Fig. S1*A*), indicating that nutrient composition, rather than calorie amount, regulates erebosis. We thus pursued the effects of individual nutrients in more detail. To evaluate the degree of erebosis, we categorized the gut tissue into three classes based on the number of erebotic cells in the R4 region. We focused on the number of GFP- nlsRFP+enterocytes, as this state represents the most frequent early/intermediate stage of erebosis (3). The extent of erebosis was classified as follows (Fig. 1*A*): class 0, three or fewer erebotic cells per field; class 1, intermediate (between class 0 and class 2); and class 2, many erebotic cells (>30% of all enterocytes).

We first focused on the sugar content. Varying sucrose concentrations in the food from 5 to 15% did not affect the frequency of erebosis significantly (Fig. 1*B*). The main component of yeast is amino acids, thus we next manipulated amino acid amounts. As the standard concentration of amino acids, we used total 296 mM amino acids, which was determined based on the concentration used in the chemically defined holidic medium (10–13). Lowering the amino acid concentration to one-tenth of the holidic medium promoted erebosis, whereas doubling it suppressed erebosis (Fig. 1*C*). We confirmed that the GFP-negative cells were labeled by Ance (Fig. 1*D*), a marker of erebosis (3), indicating that they indeed represent erebotic cells.

For these experiments, we used female flies. We found a similar effect of amino acids on erebosis in male guts (Fig. 1*E*). Amino acids also had comparable effects on enterocyte erebosis under sterile conditions (Fig. 1*F*). In this study, we mainly used female flies reared under conventional conditions. Different concentrations of amino acids affected Insulin and Tor signaling (14–19) and expression of CCHa1, a high protein-responsive gene (20) (*SI Appendix*, Fig. S1*B*), in the gut, as well as the total protein content of whole flies (*SI Appendix*, Fig. S1*C*), indicating that the synthetic diet elicited physiologically relevant effects in organisms.

First we checked involvement of autophagy in erebosis induced by low amino acid levels, since amino acid reduction often induces autophagy (21). Previously, we demonstrated that erebosis under normal food conditions does not involve autophagy (3). Erebosis induced by low amino acid concentrations did not involve autophagosome formation, detected by mCherry-Atg8a (*SI Appendix*, Fig. S2*A*). Knockdown of Atg8a or mitf, a master regulator of autophagy (22), did not affect erebosis either (*SI Appendix*, Fig. S2 *B* and *C*). This indicates that the regulatory mechanism of erebosis induced by low amino acid levels does not involve autophagy, similar to erebosis that occurs under the normal food condition.

As a sensing mechanism for amino acid shortage, the GCN2–ATF4 pathway is well established (23, 24): GCN2 is activated in response to uncharged tRNAs, leading to translation of ATF4. GCN2 can be activated by a 70% reduction in the level of single amino acids in the diet (25). ATF4 (crc) is activated when the yeast content is reduced from 10 to 4% (26). However, we found that inhibition of neither GCN2 nor ATF4 by previously validated RNAi constructs (26) suppressed erebosis induced by low amino acid levels (*SI Appendix*, Fig. S2 *D* and *E*). The independence from GCN2 and ATF4 was unexpected, as the GCN2–ATF4 pathway is typically involved in amino acid shortage sensing, with even the deprivation of a single amino acid activating GCN2. This prompted us to investigate which amino acids are specifically involved in amino acid–mediated control of erebosis.

**Fig. 2.**
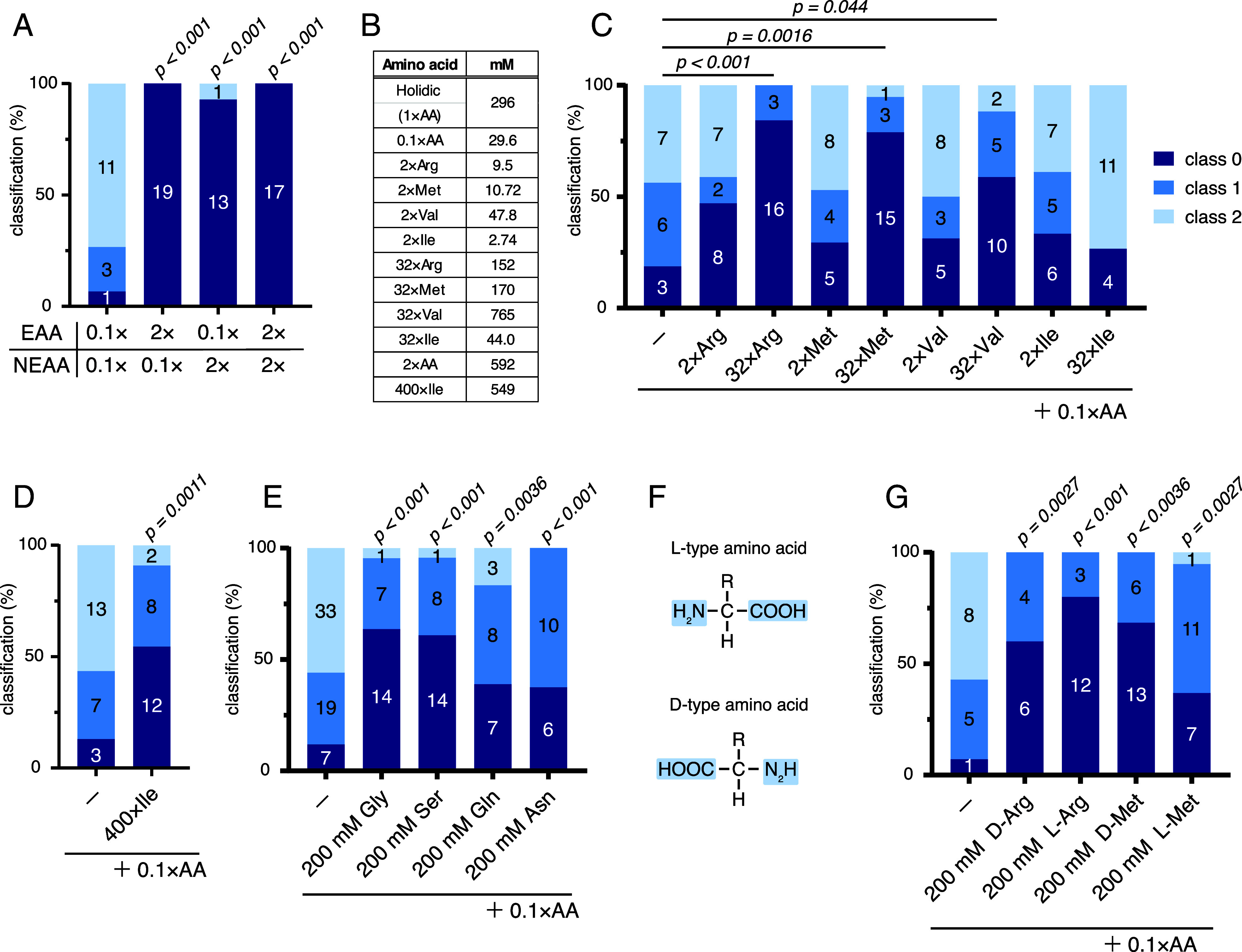
Regardless of amino acid type, single amino acids affect erebosis at high concentrations. (*A*) Either essential or nonessential amino acids suppress erebosis when provided at high concentrations. (*B*) Table showing amino acid concentrations. “1×” indicates the concentrations used in the holidic medium. (*C*) Arginine, methionine, or valine suppresses erebosis at 32× concentrations, whereas isoleucine does not. (*D*) Increasing the isoleucine concentration to 400× suppresses erebosis. (*E*) Glycine, serine, glutamine, or asparagine suppresses erebosis at 200 mM. (*F*) Chemical structures of L-amino acids and D-amino acids. (*G*) D-amino acids can also suppress erebosis similarly to L-types. *P* values were calculated using Fisher’s exact test and adjusted with the Benjamini–Hochberg method for multiple comparisons.

We examined the effects of essential and nonessential amino acids on erebosis. Both essential and nonessential amino acids suppressed erebosis (Fig. 2*A*). We then tested the effects of individual amino acids. If a specific amino acid were responsible for erebosis regulation, its concentration in the high-amino acid food used previously should suppress erebosis. However, none of the individual amino acids at the concentrations present in the high-amino acid food suppressed erebosis (Fig. 2 *B* and *C*), implying that no single amino acid is responsible for the suppression mediated by high amino acids. We increased the concentration of each single amino acid up to 32-fold that in the holidic medium. This increase suppressed erebosis (Fig. 2 *B* and *C*). Only isoleucine failed to suppress erebosis even at the 32-fold concentration (Fig. 2*C*). We noticed that the concentration of isoleucine was relatively low compared with other amino acids (Fig. 2*B*). When we further increased the amount of isoleucine, this treatment also suppressed erebosis (Fig. 2*D*).

These data suggest that increasing the concentration of single amino acids can suppress erebosis. Approximately 200 mM of single amino acids was sufficient to inhibit erebosis. We thus fed flies with 200 mM of other amino acids, glycine, serine, glutamine, or asparagine, all of which suppressed erebosis (Fig. 2*E*). We also found that D-amino acids, which do not constitute proteins in contrast to protein-forming L-amino acids (27), suppressed erebosis as well (Fig. 2 *F* and *G*), suggesting that protein synthesis is not involved in amino acid-mediated regulation of erebosis. In sum, there was no amino acid specificity in the regulation of erebosis.

**Fig. 3.**
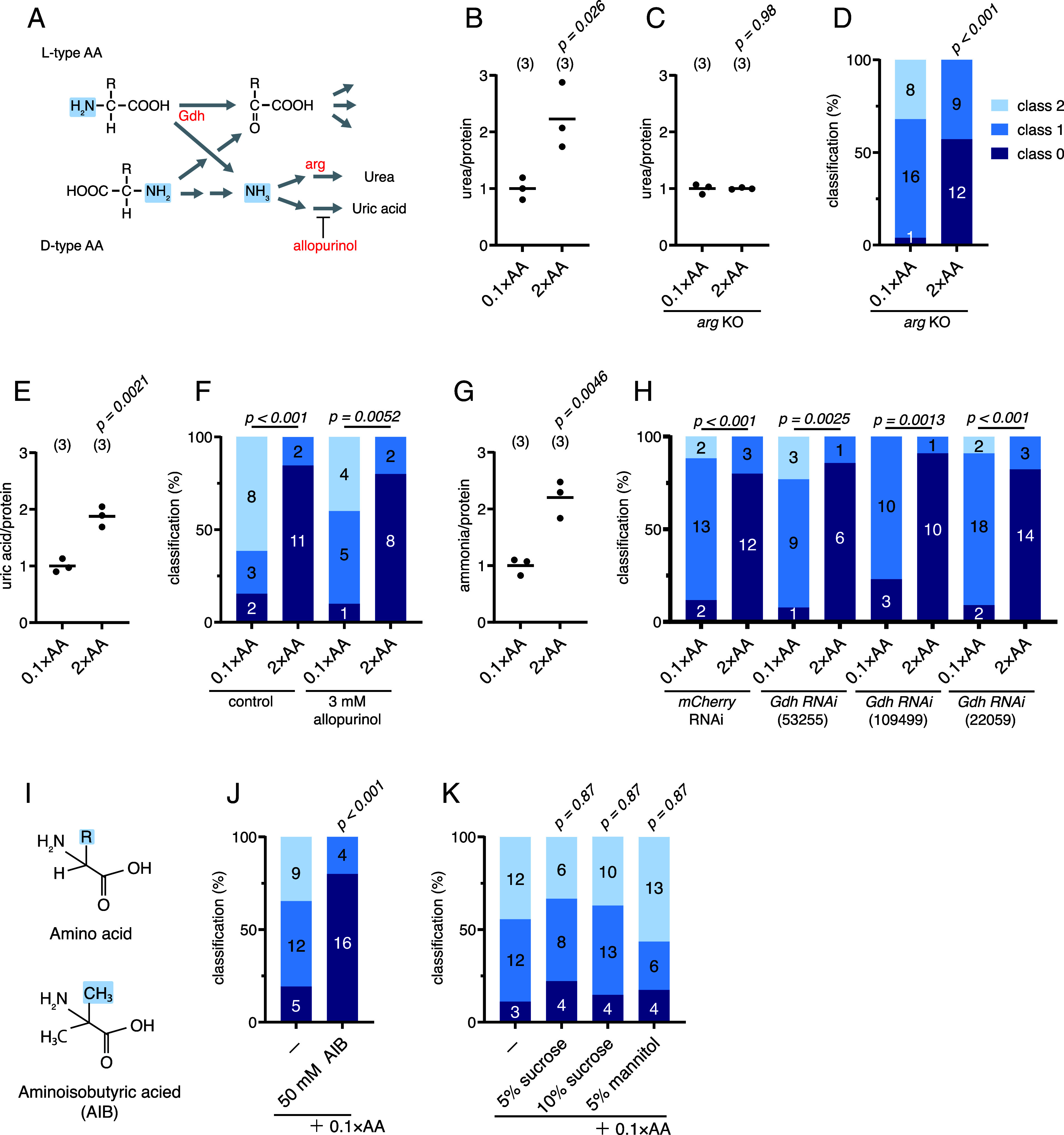
Amino acid metabolism is dispensable for the effects of amino acids on erebosis. (*A*) As final products of amino acid catabolism, urea and uric acid are generated from ammonia. (*B*) High concentrations of amino acids increase urea levels in flies. (*C*) Arginase knockout inhibits the amino acid-induced increase of urea. (*D*) High concentrations of amino acids suppress erebosis in the arginase knockout mutant. (*E*) High concentrations of amino acids increase uric acid levels in flies. (*F*) Inhibition of uric acid production does not suppress the effects of amino acids on erebosis. (*G*) High concentrations of amino acids increase ammonia levels in flies. (*H*) Inhibition of ammonia production by Gdh RNAi does not suppress the effects of amino acids on erebosis. (*I*) Structural formulas of conventional amino acid and amino acid analog AIB. (*J*) The unmetabolizable amino acid analog AIB suppresses erebosis at a relatively low concentration (50 mM). (*K*) The cell-impermeable compound mannitol does not affect erebosis. *P* values were calculated using the unpaired *t* test (*B*, *C*, *E*, and *G*) or Fisher’s exact test (*D*, *F*, *H*, *J*, and *K*) and adjusted with the Benjamini–Hochberg method for multiple comparisons (*K*).

Since all amino acids examined, regardless of their type, including non–protein-forming D-amino acids, could suppress erebosis, we hypothesized that a common feature among amino acids might regulate erebosis. We focused on the final products of amino acid metabolism, urea, and uric acid (Fig. 3*A*). Indeed, high–amino acid food increased the levels of urea and uric acid (Fig. 3 *B* and *E*), but inhibition of either, via arginase knockout (28) or pharmacological suppression of uric acid (29), did not block the amino acid-mediated suppression of erebosis (Fig. 3 *C*, *D*, and *F*). We also examined ammonia, which was similarly elevated by high–amino acid food (Fig. 3*G*). However, inhibition of ammonia production by suppressing glutamate dehydrogenase, a key enzyme for generating ammonia from glutamate produced by transamination (30, 31), either through genetic knockdown or a specific inhibitor, did not prevent the suppression of erebosis either (Fig. 3*H* and *SI Appendix*, Fig. S3 *A* and *B*).

Up to this point, we demonstrated that any amino acid examined, regardless of its type, can suppress erebosis, independently of its metabolism to urea, uric acid, or ammonia. We hypothesized that metabolism may not be required for amino acid-mediated regulation of erebosis. To test this, we examined the effects of α-aminoisobutyrate, an inert bioorthogonal amino acid analog that cannot be metabolized but can enter cells through amino acid transporters (Fig. 3*I*). α-aminoisobutyrate is often used as a tracer for amino acid transport activity (32, 33). We found that α-aminoisobutyrate suppressed erebosis at a relatively low concentration (50 mM) (Fig. 3*J*), at which normal amino acids such as isoleucine could not suppress erebosis (Fig. 2 *B*–*D*). This indicates that amino acid metabolism is not required for regulation of erebosis. It also reinforces our finding that urea, uric acid, or ammonia is not involved in erebosis.

What do amino acids affect in cells without being metabolized? We considered the possible involvement of extracellular osmolarity, but this seemed unlikely. Increasing the concentration of sucrose did not affect erebosis (Fig. 3*K*), whereas relatively low concentrations of the bioorthogonal molecule α-aminoisobutyrate strongly suppressed it, suggesting that this effect is not due to changes in extracellular osmolarity. Consistently, treatment with the cell-impermeable osmolyte mannitol did not alter erebosis (Fig. 3*K*). Most likely, enterocytes, which are constantly exposed to fluctuating luminal environments, are protected from osmotic changes by the peritrophic membrane and other apical barrier structures such as glycocalyx (34, 35).

**Fig. 4.**
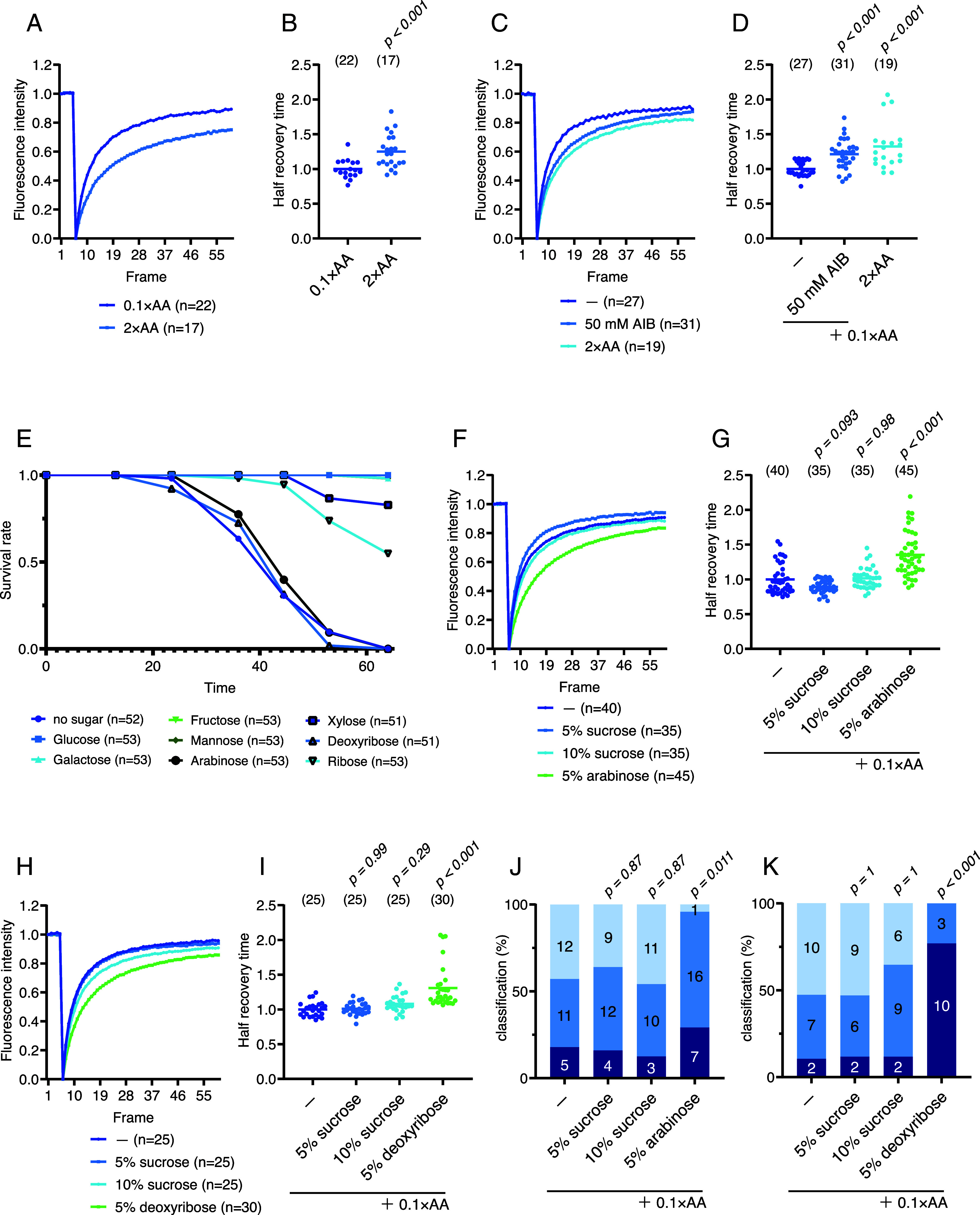
High amino acids and three independent viscogens alter effective cytoplasmic viscosity detected by GFP FRAP and affect erebosis. (*A*) Concentrations of amino acids in the food affect recovery of GFP after photobleaching in enterocytes. (*B*) Concentrations of amino acids in the food affect the GFP half-recovery time. (*C*) The unmetabolizable amino acid analog AIB slows GFP recovery similarly to 2× AA. (*D*) AIB and 2× AA exhibit similar effects on the GFP half-recovery time. (*E*) Survival curves under complete starvation indicate that flies can utilize fructose, xylose, glucose, mannose, galactose, and ribose as energy sources, but not deoxyribose or arabinose. (*F*) Feeding arabinose slows GFP recovery. (*G*) Arabinose lengthens the GFP half-recovery time after photobleaching. (*H*) Feeding deoxyribose slows GFP recovery. (*I*) Deoxyribose lengthens the GFP half-recovery time after photobleaching. (*J*) Feeding arabinose suppresses enterocyte erebosis. (*K*) Feeding deoxyribose suppresses enterocyte erebosis. *P* values were calculated using the unpaired *t* test (*B*), ANOVA with Dunnett’s multiple comparison (*D*, *G*, and *I*) or Fisher’s exact test and adjusted with the Benjamini–Hochberg method for multiple comparisons (*J* and *K*).

Thus far, we demonstrated that the quantity rather than the quality of amino acids is important for controlling erebosis, and that even the nonmetabolizable amino acid analog can regulate it. We inferred that the principal and common effect of amino acids, or their inert analog, might be on a biophysical property within cells, specifically, cytoplasmic fluidity. Cytoplasmic fluidity is particularly influenced by intracellular viscosity, which reflects molecular crowding of both macromolecules such as ribosomes and small molecules such as trehalose and sorbitol (36–40). To analyze cytoplasmic fluidity, we performed FRAP analysis of GFP, which is ~4 nm in diameter, under different dietary conditions. Although FRAP-based measurements of fluorescent tracer mobility have been widely used to estimate effective intracellular diffusion, such measurements reflect not only solvent viscosity but also molecular crowding, obstruction, and transient interactions (41–43). Thus, FRAP reflects effective cytoplasmic viscosity, which is operationally defined as the apparent resistance experienced by a diffusing GFP molecule within the cytoplasm (41–43). Consistent with our hypothesis, high amino acid levels increased effective viscosity detected by FRAP of GFP (Fig. 4 *A* and *B*), as did α-aminoisobutyrate (Fig. 4 *C* and *D*). These findings led us to think that nutritional status affects cytoplasmic fluidity, which in turn influences erebosis.

If cytoplasmic fluidity is critical for erebosis, its regulator does not necessarily have to be amino acids. We demonstrated that sucrose, which is broken down into glucose and fructose, does not affect erebosis (Figs. 1*B* and 3*K*), likely because cells metabolize glucose and fructose rapidly and maintain intracellular glucose/fructose concentrations within a narrow range. We sought to manipulate cellular fluidity independently of amino acids. If altering cytoplasmic fluidity through methods other than amino acids or α-aminoisobutyrate also affects erebosis, this would support the idea that cytoplasmic fluidity itself regulates erebosis.

We searched for bioorthogonal viscogens that could enter cells but remain metabolically inert in flies. To identify small molecule viscogens that flies cannot metabolize, we utilized an organismal starvation assay. In this assay, if flies can use a molecule as an energy source through its metabolism, the compound provides a survival advantage under starvation. Using this approach, we found that flies could utilize glucose, fructose, galactose, mannose, xylose, and ribose as energy sources, but not arabinose or deoxyribose (Fig. 4*E*). It is noteworthy that arabinose can enter *Drosophila* cells (44), and deoxyribose is likely transported into cells through the same transporter with ribose, such as the glucose transporter family (45). We considered arabinose and deoxyribose to be metabolically inert and used them to manipulate effective cytoplasmic viscosity.

We found that feeding arabinose and deoxyribose made the enterocyte cytoplasm more viscous, that is, less fluid based on the GFP FRAP (Fig. 4 *F*–*I*). We then examined the effects of these independent viscogens on erebosis. Remarkably, these completely different types of compounds suppressed erebosis (Fig. 4 *J* and *K*). The biochemical properties of the three viscogens, α-aminoisobutyrate, arabinose, and deoxyribose, are highly distinct, yet all of them suppressed erebosis. Contrary to feeding of free amino acids (*SI Appendix*, Fig. S1*B*), effects of the viscogens on the conventional nutrient signaling such as Insulin and Tor pathways were negligible (*SI Appendix*, Fig. S4), indicating that these affect erebosis independent on the conventional nutrient sensing pathways. These findings strongly support the idea that cytoplasmic fluidity serves as a sensor that converts nutritional status into the regulation of erebosis.

**Fig. 5.**
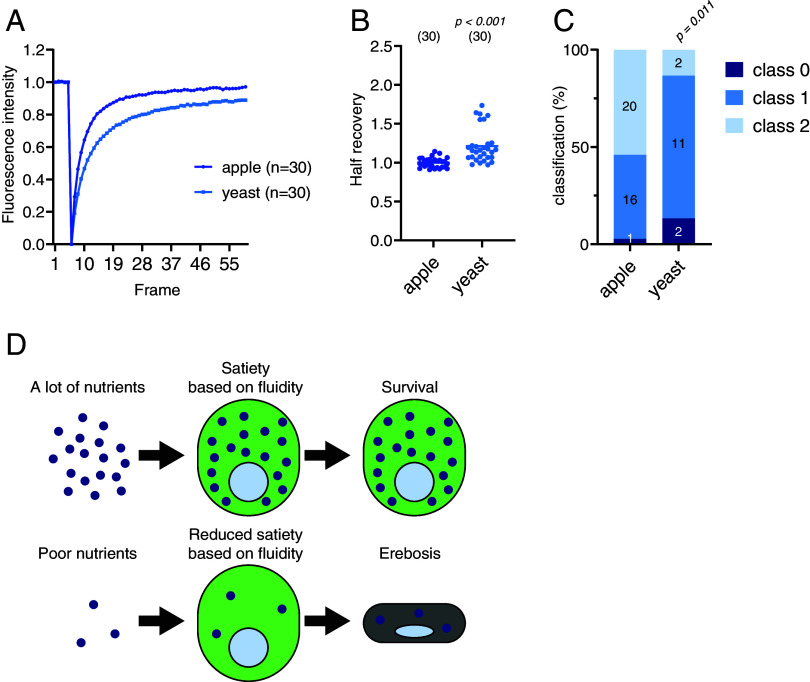
Physiological relevance of fluidity-mediated regulation of erebosis. (*A*) Feeding apples, a low-protein fruit, promotes GFP recovery after photobleaching compared with feeding yeast, a high-protein diet. (*B*) Feeding yeast lengthens the GFP half-recovery time. (*C*) Apple-fed flies show more erebotic cells in the gut than yeast-fed flies. (*D*) Detection of nutrient availability based on cytoplasmic fluidity. Enterocytes sense hunger and satiety through cytoplasmic fluidity. When food is abundant and cytoplasmic fluidity remains low, enterocytes survive to absorb nutrients. When food becomes scarce and cytoplasm becomes more fluid, enterocytes undergo erebosis as an adaptive response. *P* values were calculated using the unpaired *t* test (*B*) or Fisher’s exact test (*C*).

We also examined the physiological relevance of cytoplasmic fluidity-based detection of dietary components. Fruit flies are known to feed on both fruits and yeast in the wild, and fruits generally contain less protein than yeast. We found that these natural diets exerted opposite effects on cytoplasmic fluidity and erebosis: Yeast decreased cytoplasmic fluidity, as detected by FRAP, and suppressed erebosis compared with apples (Fig. 5 *A*–*C*). Thus, our model that erebosis is regulated by diet-mediated cytoplasmic fluidity can be applied to a physiologically relevant situation.

## Discussion

A traditional view holds that cells sense nutritional status primarily through biochemical pathways that are selectively activated by distinct nutrients. Beyond this conventional framework, recent work in yeast and cultured mammalian cells suggests that nutrients can also affect the biophysical property of the cytoplasm, altering fluidity and crowding (37, 46). Yet whether such changes constitute a physiologically relevant in vivo signal within multicellular organisms has remained unclear. Here, we propose that intracellular fluidity provides a previously missing, physical layer of nutrient detection in the gut epithelium. In our data, the quantity of amino acids, rather than their quality, governs enterocyte fate by modulating cytoplasmic fluidity and thereby influencing the frequency of erebosis.

It is of note that our FRAP measurements monitor the diffusional recovery of cytoplasmic GFP (~4 nm hydrodynamic diameter), thereby reporting changes in effective nanoscale microviscosity experienced by small cytosolic proteins. The regime of GFP diffusion is distinct from mesoscale crowding, which primarily reflects crowding of macromolecules caused by ribosomes and large protein complexes (37). The diffusion of small molecules such as GFP is largely insensitive to mesoscale crowding, which instead affects the motion of 20 to 40 nm particles such as GEMs. Previous studies often linked nutrient availability to mesoscale crowding but not to nanosclae microviscosity (37, 46). In contrast, our observation that both L- and D-amino acids, as well as metabolically inert small molecules, decrease the apparent nanosclae fluidity detected by GFP FRAP suggests that small solutes themselves can tune the physical state of the cytoplasm.

What is the molecular mechanism that links cytoplasmic fluidity and erebosis? Cytoplasmic fluidity can influence phase transitions. Crowding sensors such as WNK kinases are known to be regulated by phase separation dynamics (47). Thus, there may exist a general viscosity sensor that detects cellular viscosity or fluidity through liquid–liquid phase separation. Alternatively, although the molecular mechanism of erebosis remains elusive, the core machinery of erebosis itself may be directly affected by changes in cytoplasmic fluidity. To clarify how cells sense cytoplasmic fluidity and convert this physical cue into the execution of erebosis is the most important, urging future question we will pursue.

Erebosis is a process toward cell death in which cells become flattened and enlarged (3). Although all enterocytes are postmitotic and the conventional definition of cellular senescence based on cell cycle arrest (48)cannot be directly applied to erebosis, the flattened and enlarged morphology is reminiscent of senescent cells. It is intriguing to note that cytoplasmic dilution has been proposed to contribute to cellular senescence in yeast (49). Because cytoplasmic dilution is expected to increase cellular fluidity, there might be common underlying mechanisms between erebosis and yeast replicative senescence.

Regarding physiological relevance of our findings, regulating cellular state based on cytoplasmic fluidity can allow cells to respond to the overall intracellular milieu, which reflects the combined concentrations of virtually all molecules in the cell. This integrated cellular environment is shaped by both the abundance and metabolic turnover of individual dietary components. Whether the fluidity-based response to metabolites occurs in cell types other than enterocytes in vivo remains unclear. For enterocytes, which are directly exposed to a constantly changing, wide variety of dietary components, sensing the nutritional environment through cytoplasmic fluidity may represent a particularly appropriate strategy.

We propose that fluidity-mediated sensing of dietary components constitutes an adaptive mechanism that adjusts cell fate according to nutrient availability (Fig. 5*D*). When nutrients are abundant, enterocytes can be maintained to support active nutrient absorption. Under nutrient-poor conditions, however, maintaining a large population of enterocytes may become metabolically inefficient because of both the limited nutrient supply and the energetic cost of sustaining excess cells. Thus, the induction of erebosis under nutrient-poor conditions may represent an adaptive strategy for optimizing tissue homeostasis.

## Methods

### Fly Husbandry.

Flies were maintained as previously described (50). They were kept on standard fly food containing 0.8% agar, 10% glucose, 4.5% corn flour, 3.72% dry yeast, 0.4% propionic acid, 0.3% butyl p-hydroxybenzonate. Crosses and experiments were conducted at 25 °C unless otherwise specified.

### *Drosophila* Stocks.

The following stocks were used in this study:

w; Myo1D-Gal4, UAS-GFP, UAS-nlsRFP; tub-gal80ts

w; Myo1D-Gal4, UAS-GFP, UAS-nlsRFP/Cyo

w; Myo1D-Gal4, UAS-GFP/Cyo

mCherry Atg8a (51)

mCherry RNAi (BDSC 35785)

Atg8a RNAi (BDSC 28989)

Mitf RNAi (BDSC 34835)

Mitf RNAi (BDSC 43998)

Mitf RNAi (BDSC 44561)

GCN2 RNAi (BDSC 67215)

GCN2 RNAi (VDRC v103976)

Arg KO (BDSC 59660)

Gdh RNAi (BDSC 53255)

Gdh RNAi (VDRC v109499)

Gdh RNAi (VDRC v22059)

crc RNAi (VDRC v109014)

crc RNAi (BDSC 80388)

### Food Conditions.

A few days old virgin female flies were collected and maintained for a week on the following conditions.

*SI Appendix*, Fig. S1*A*: high-yeast low-sugar food (0.8% agar, 5% sucrose, 10% dry yeast, 0.4% propionic acid, 0.3% butyl p-hydroxybenzoate), intermediate food (0.8% agar, 10% sucrose, 3.5% dry yeast, 0.4% propionic acid, 0.3% butyl p-hydroxybenzoate), high-sugar low yeast food (0.8% agar, 12.5% sucrose, 0.35% dry yeast, 0.4% propionic acid, 0.3% butyl p-hydroxybenzoate).

Fig. 1*B*: low-sucrose food (1.5% agar, 10% peptone, 5% sucrose, 0.3% propionic acid, 0.3% butyl p-hydroxybenzoate), intermediate sucrose food (1.5% agar, 10% peptone, 10% sucrose, 0.3% propionic acid, 0.3% butyl p-hydroxybenzoate), or high-sucrose food (1.5% agar, 10% peptone, 15% sucrose, 0.3% propionic acid, 0.3% butyl p-hydroxybenzoate).

For amino acid feeding, 1× AA was calculated based on the holidic medium (10, 11): 1.5% agar, 10% sucrose, 0.3% propionic acid, 0.3% butyl p-hydroxybenzoate, and the following amino acids: 4.75 mM L-arginine hydrochloride, 23.90 mM L-valine, 1.37 mM L-isoleucine, 0.91 mM L-leucine, 5.36 mM L-methionine, 10.40 mM L-lysine, 7.87 mM L-phenylalanine, 2.45 mM L-tryptophan, 16.79 mM L-threonine, 6.45 mM L-histidine, 13.03 mM L-proline, 42.63 mM glycine, 39.29 mM L-alanine, 0.41 mM L-cysteine, 18.08 mM L-serine, 0.22 mM L-tyrosine, 12.87 mM L-asparagine, 12.77 mM L-aspartic acid, 17.11 mM L-glutamine, and 59.13 mM L-glutamic acid), low-amino acid food (1.5% agar, 10% sucrose, 0.3% propionic acid, 0.3% butyl p-hydroxybenzoate, and one-tenth the amino acid concentration of the holidic medium-based food), or high-amino acid food (1.5% agar, 10% sucrose, 0.3% propionic acid, 0.3% butyl p-hydroxybenzoate, and twice the amino acid concentration of the holidic medium-based food).

Fig. 2*A*: high-EAA low-NEAA food (1.5% agar, 10% sucrose, 0.3% propionic acid, 0.3% butyl p-hydroxybenzoate, and twice the concentration of the 10 essential amino acids and one-tenth the concentration of the 10 nonessential amino acids relative to the holidic medium-based food), or high-NEAA low-EAA food (1.5% agar, 10% sucrose, 0.3% propionic acid, 0.3% butyl p-hydroxybenzoate, and twice the concentration of the 10 nonessential amino acids and one-tenth the concentration of the 10 essential amino acids relative to the holidic medium-based food).

Single amino acid feeding: L-arginine (4.75, 152, or 200 mM), L-methionine (5.36, 170, or 200 mM), L-valine (23.9 or 765 mM), L-isoleucine (1.37, 44.0, or 549 mM), glycine (200 mM), L-serine (200 mM), L-glutamine (200 mM), L-asparagine (200 mM), D-arginine (200 mM), or D-methionine (200 mM).

### Antibiotic Treatment.

Eggs were collected on grape plates with live yeast paste for 24 h. To remove bacteria, eggs were dechorionated in PURELOX-S (containing 6% hypochlorous acid; OYALOX Co., Ltd.) for 90 s and then transferred onto antibiotic-containing 1× SY food (1.5% agar, 5% sucrose, 10% yeast, 0.3% propionic acid, 0.3% butyl p-hydroxybenzoate, and 1% penicillin-streptomycin solution; Wako 168-23191). For the control, eggs were washed with water and transferred onto 1× SY food without antibiotics. Virgin female flies were collected and maintained for 7 d on one of the following diets: antibiotic-containing low-amino acid food (1.5% agar, 10% sucrose, 0.3% propionic acid, 0.3% butyl p-hydroxybenzoate, one-tenth the amino acid concentration of the holidic medium-based food, and 1% penicillin-streptomycin solution), antibiotic-containing high-amino acid food (1.5% agar, 10% sucrose, 0.3% propionic acid, 0.3% butyl p-hydroxybenzoate, twice the amino acid concentration of the holidic medium-based food, and 1% penicillin-streptomycin solution), low-amino acid food (1.5% agar, 10% sucrose, 0.3% propionic acid, 0.3% butyl p-hydroxybenzoate, and one-tenth the amino acid concentration of the holidic medium-based food), or high-amino acid food (1.5% agar, 10% sucrose, 0.3% propionic acid, 0.3% butyl p-hydroxybenzoate, and twice the amino acid concentration of the holidic medium-based food).

### Confocal Imaging.

After feeding, guts were dissected in 1× PBS, fixed for 60 min at RT in PBS with 4% paraformaldehyde (PFA, Thermo Fisher Scientific, 43368), and washed by PBSTx for three times. They were incubated with PBSTx with Hoechst (Thermo Fisher Scientific H1399) at 4 °C overnight. After the incubation, guts were washed in PBSTx three times, mounted, and observed. Fluorescent images of the posterior midgut (R4) were acquired using a LSM 900 (Zeiss) confocal microscope at 20× or 40× magnification.

### Immunohistochemistry.

After feeding, guts were dissected in 1× PBS, fixed for 60 min at RT in PBS with 4% paraformaldehyde, and washed by PBSTx for three times. They were incubated with PBSTx with 10% normal goat serum (NGS) and a primary antibody at 4 °C overnight, followed by washing in PBSTx three times and incubation with PBSTx with 10% NGS, 100 μg/mL Hoechst, and secondary antibody at 4 °C overnight. After the incubation, guts were washed in PBSTx five times.

Antibodies and their dilutions are as follows:

Rabbit anti-Ance 1:1,000 (A gift from Elwyn Isaac, University of Leeds, UK).

Goat anti-rabbit IgG Alexa Fluor 633 1:500 (Thermo Fisher Scientific A21070).

### RNAi.

For crc RNAi experiments, w-; Myo1D-Gal4, UAS-GFP, UAS-nlsRFP; tub-Gal80ts virgin female flies were crossed with male RNAi flies at 18 °C. Newly eclosed virgin female flies were collected and briefly kept at 18 °C, then transferred to 30 °C to induce RNAi expression for 3 d.

After the induction, flies were kept at 25 °C for 4 d and fed with either low-amino acid food (1.5% agar, 10% sucrose, 0.3% propionic acid, 0.3% butyl p-hydroxybenzoate, and one-tenth the amino acid concentration of the holidic medium-based food) or high-amino acid food (1.5% agar, 10% sucrose, 0.3% propionic acid, 0.3% butyl p-hydroxybenzoate, and twice the amino acid concentration of the holidic medium-based food).

For other RNAi experiments, w-; Myo1D-Gal4, UAS-GFP, UAS-nlsRFP virgin female flies were crossed with male RNAi flies at 25 °C. Newly eclosed virgin female flies were collected and kept at 25 °C for 7 d and fed with either low-amino acid food (1.5% agar, 10% sucrose, 0.3% propionic acid, 0.3% butyl p-hydroxybenzoate, and one-tenth the amino acid concentration of the holidic medium-based food) or high-amino acid food (1.5% agar, 10% sucrose, 0.3% propionic acid, 0.3% butyl p-hydroxybenzoate, and twice the amino acid concentration of the holidic medium-based food).

### Ammonia Measurement.

After feeding, five flies were homogenized in 100 µL PBSTx in a 1.5 mL microtube and centrifuged at 4 °C and 10,000 rpm for 4 min. The resulting supernatant was used for the assay. Ammonia levels were measured according to the manufacturer’s protocol of the Ammonia Assay Kit (Sigma, AA0100). In this assay, ammonia reacts with α-ketoglutarate and NADPH in the presence of L-glutamate dehydrogenase (GDH) to produce L-glutamate and NADP^+^. The oxidation of NADPH causes a decrease in absorbance at 340 nm, which is proportional to the ammonia concentration. The amount of ammonia was determined by measuring the change in absorbance at 340 nm before and after addition of GDH. Ammonia concentrations were normalized to the total protein concentration of each sample. Total protein concentrations were determined using a BCA Protein Assay Kit (Thermo Fisher, Pierce 23227), and absorbance at 560 nm was measured with a multimode plate reader.

### Quantification of RNAi Efficiency.

RNA was extracted from three L3 stage larvae per sample by using the Maxwell RSC simplyRNA Tissue Kit (Promega). RNA was subjected to DNase digestion, followed by reverse transcription using 5× prime script RT master mix (Takara). qPCR was performed using the FastStart Essential DNA Green Master Mix (Roche). Rpl32 was used as an internal control. RNAi efficiency was determined by comparing the relative expression levels (2^^−ΔΔCq^ values) between the RNAi and control.

Primers used for qPCR are as follows:

RpL32 forward primer: 5′-CCAGCATACAGGCCCAAGATCGTG-3′

RpL32 reverse primer: 5′-TCTTGAATCCGGTGGGCAGCATG-3′

Gdh forward primer: 5′- GCTGCCGTCGAGAAGGTCATC-3′

Gdh reverse primer: 5′- CCACTCGAAGAAGGAGACGGTGA-3′

### Drug Feeding.

Virgin female flies were collected and maintained for 7 d on either low-amino acid food (1.5% agar, 10% sucrose, 0.3% propionic acid, 0.3% butyl p-hydroxybenzoate, and one-tenth the amino acid concentration of the holidic medium-based food) or high-amino acid food (1.5% agar, 10% sucrose, 0.3% propionic acid, 0.3% butyl p-hydroxybenzoate, and twice the amino acid concentration of the holidic medium-based food) supplemented with the R162 stock solution to yield final concentrations of 0, 250 µM, 500 µM, or 1 mM. For controls, the same volume of DMSO was added.For allopurinol treatment, virgin female flies were collected and maintained for 7 d on one of the following diets: low-amino acid food (1.5% agar, 10% sucrose, 0.3% propionic acid, 0.3% butyl p-hydroxybenzoate, and one-tenth the amino acid concentration of the holidic medium-based food), high-amino acid food (1.5% agar, 10% sucrose, 0.3% propionic acid, 0.3% butyl p-hydroxybenzoate, and twice the amino acid concentration of the holidic medium-based food), low-amino acid with allopurinol (Fujifilm Wako) food (1.5% agar, 10% sucrose, 0.3% propionic acid, 0.3% butyl p-hydroxybenzoate, 3 mM allopurinol, and one-tenth the amino acid concentration of the holidic medium-based food), or high-amino acid with allopurinol food (1.5% agar, 10% sucrose, 0.3% propionic acid, 0.3% butyl p-hydroxybenzoate, 3 mM allopurinol, and twice the amino acid concentration of the holidic medium-based food).

### LC/MS Analysis of Urea and Uric Acid from *Drosophila*.

Three flies were transferred into a 1.5 mL microtube and frozen at −80 °C. A zirconia bead (φ3 mm) and 300 µL of chilled methanol were added, and the samples were homogenized using a Freeze Crusher μT-48 (TAITEC) at room temperature and 2,500 rpm for 2 min. 400 µL of chilled 50% methanol and 200 µL of chloroform were sequentially added and mixed. The mixture was further agitated for 10 min using a Microtube Mixer MT-400 (TOMY) and centrifuged at 4 °C and 15,000 rpm for 10 min. The resulting supernatant (800 µL) was transferred to a new tube, mixed with 400 µL of water, and centrifuged again under the same conditions. The collected supernatant was divided into two tubes (400 µL each) and dried at room temperature using a centrifugal concentrator VA-500R (TAITEC) at 1,500 rpm for 4 h. The dried extracts were dissolved in 80 µL of 50% acetonitrile, combined, and centrifuged again at 4 °C and 15,000 rpm for 10 min. A 2 µL aliquot of the final supernatant was subjected to LC/MS analysis.

The extracts were analyzed using a liquid chromatography system (Waters, ACQUITY UPLC H-Class) coupled to a tandem quadrupole mass spectrometer (Waters, XEVO TQ-S).A standard curve was generated using a urea standard (Nacalai Tesque, 35907-15), and the concentrations of urea and uric acid in the samples were quantified based on peak intensities using MassLynx software (Waters, Ver. 4.1). Finally, the LC/MS based concentrations of urea and uric acid were normalized to the total protein concentration of each sample.Total protein concentrations were determined using a BCA Protein Assay Kit, and absorbance at 560 nm was measured with a multimode plate reader.

### AIB Feeding.

Virgin female flies were collected and maintained for 7 d on one of the following diets: low-amino acid food (1.5% agar, 10% sucrose, 0.3% propionic acid, 0.3% butyl p-hydroxybenzoate, and one-tenth the amino acid concentration of the holidic medium-based food), or AIB (Merck 850993) containing food (1.5% agar, 10% sucrose, 0.3% propionic acid, 0.3% butyl p-hydroxybenzoate, 50 mM α-aminoisobutyric acid, and one-tenth the amino acid concentration of the holidic medium-based food).

### Mannitol Feeding.

Virgin female flies were collected and maintained for 7 d on one of the following diets: normal-sucrose food (1.5% agar, 10% sucrose, 0.3% propionic acid, 0.3% butyl p-hydroxybenzoate, and one-tenth the amino acid concentration of the holidic medium-based food), high-sucrose food (1.5% agar, 15% sucrose, 0.3% propionic acid, 0.3% butyl p-hydroxybenzoate, and one-tenth the amino acid concentration of the holidic medium-based food), very high-sucrose food (1.5% agar, 20% sucrose, 0.3% propionic acid, 0.3% butyl p-hydroxybenzoate, and one-tenth the amino acid concentration of the holidic medium-based food), or mannitol-containing food (1.5% agar, 10% sucrose, 5% mannitol, 0.3% propionic acid, 0.3% butyl p-hydroxybenzoate, and one-tenth the amino acid concentration of the holidic medium-based food).

### FRAP Analysis.

After feeding, guts were dissected in 1× PBS and immediately mounted in 1× PBS for live imaging. FRAP analysis was performed on the posterior midgut (R4) using a LSM 900 (Zeiss) confocal microscope at 40× magnification. GFP-positive enterocytes were selected, and a cytoplasmic area (20 to 60 µm^2^) was bleached with a laser at the fifth frame. Fluorescence recovery was recorded for 60 frames. A nonbleached cytoplasmic area within the same cell (3 µm^2^) was used as a reference for intensity normalization. The fluorescence intensity of the bleached region was divided by the reference region, normalized by setting the first frame was set to 1 and the sixth frame (just after bleaching) was set to 0. The half-recovery time was defined as the frame number at which the fluorescence intensity reached half of the final frame value.

### Starvation Assay.

One-wk-old Oregon R male flies were placed on 1.5% agar containing 5% of the following monosaccharides: glucose, galactose, fructose, mannose, arabinose, xylose, deoxyribose, or ribose. Survival was recorded every 24 h.

### Arabinose/Deoxyribose Feeding.

Virgin female flies were collected and maintained for 7 d on one of the following diets: normal-sucrose food (1.5% agar, 10% sucrose, 0.3% propionic acid, 0.3% butyl p-hydroxybenzoate, and one-tenth the amino acid concentration of the holidic medium-based food), high-sucrose food (1.5% agar, 15% sucrose, 0.3% propionic acid, 0.3% butyl p-hydroxybenzoate, and one-tenth the amino acid concentration of the holidic medium-based food), very high-sucrose food (1.5% agar, 20% sucrose, 0.3% propionic acid, 0.3% butyl p-hydroxybenzoate, and one-tenth the amino acid concentration of the holidic medium-based food), arabinose (TCI A0515)-containing food (1.5% agar, 10% sucrose, 5% arabinose, 0.3% propionic acid, 0.3% butyl p-hydroxybenzoate, and one-tenth the amino acid concentration of the holidic medium-based food) or deoxyribose (TCI D0059)-containing food (1.5% agar, 10% sucrose, 5% deoxyribose, 0.3% propionic acid, 0.3% butyl p-hydroxybenzoate, and one-tenth the amino acid concentration of the holidic medium-based food).

### Protein Measurement.

Adult female flies were kept on 0.1× AA or 2× AA food for 7 d. Each of the 10 samples consisted of two female flies. They were frozen at −80 °C. Samples were homogenized in 100 μL cold PBST with a plastic pestle and kept on ice. Immediately, samples were heat-inactivated at 80 °C for 10 min using a block incubator (Astec). For each sample, 10 μL was used for total protein measurement. Protein concentration was determined using a BCA assay kit (Thermo, cat. 23227) by measuring absorbance at 562 nm with a Multimode Plate Reader (NIVO, PerkinElmer).

### RNA-seq.

Total RNA was purified with the Maxwell RSC simplyRNA Tissue Kit (Promega) from guts of female flies kept on different A.A. concentrations for 3 and 7 d (*SI Appendix*, Fig. S1*B*) or on different viscogens for 3 d (*SI Appendix*, Fig. S4). Each RNA sample was from seven guts and 3 or 5 samples were used for one condition. RNA samples were sequenced by NovaSeq 6000. Sequencing data were analyzed by CLC Genomics Workbench. Expression was calculated from TPM values.

### Apple and Yeast Feeding.

Virgin female flies were collected and maintained for 7 d on either fresh apple or yeast paste, the latter prepared by mixing dry yeast with water to form a paste. Apples were purchased at Life Kasuganomichi (Kobe).

### Statistics.

Statistical analyses were conducted by R version 4.2.0 or Prism. For multiple comparisons of Fisher’s exact test, *P*-values were corrected by the Benjamini–Hochberg method. For post hoc test ANOVA, Dunnett’s multiple comparison was performed.

## Supplementary Material

Appendix 01 (PDF)

## Data Availability

Study data are included in the article and/or *SI Appendix*.
