# Supplementary material for: Cytoplasmic fluidity couples nutrient availability and enterocyte fate in vivo
Source: Proc Natl Acad Sci U S A. 2026 Aug 4;123(32):e2602724123. doi: 10.1073/pnas.2602724123 (PMC13462174; doi:10.1073/pnas.2602724123)
Supplement: Supplementary file 1 [file pnas.2602724123.sapp.pdf]

FigS1

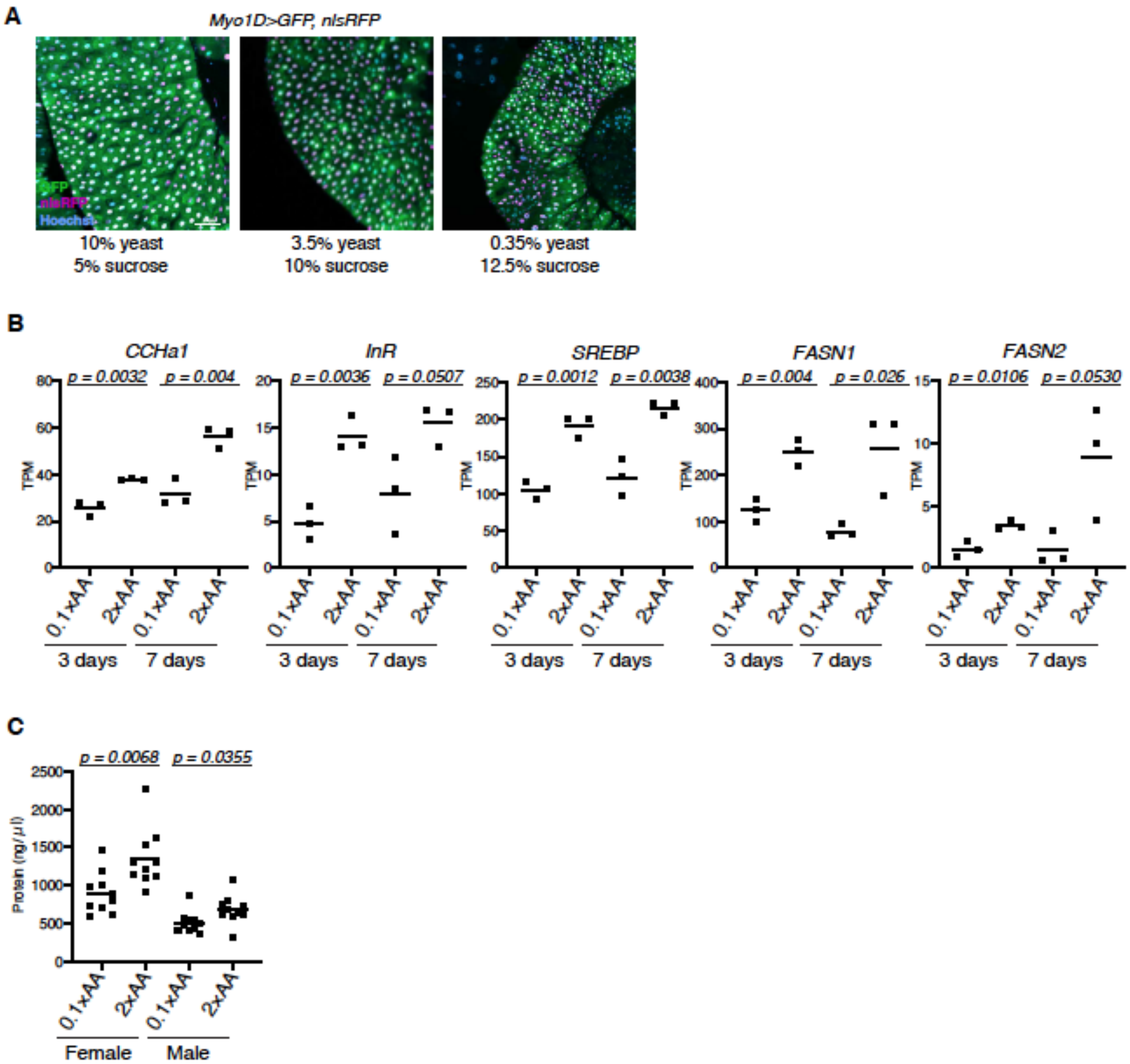

Figure S1 Effects of food components on erebosis and Insulin and Tor signaling

A. Among foods with the same caloric content, the diet low in yeast and high in sucrose induces erebosis.

B. Flies were fed with either low (0.1xAA) or high (2xAA) amino acid food for 3 or 7 days and the RNA-seq was performed using mRNA from the guts. CCHa1 is a high-protein-responsive gene, InR is regulated by Insulin signaling and SREBP/FASN1/FASN2 are regulated by Insulin and Tor signaling.

C. Whole-organism protein content was measured 7 days after flies were transferred to different amino acid conditions.

P values were calculated using unpaired t-test (B-C).

FigS2

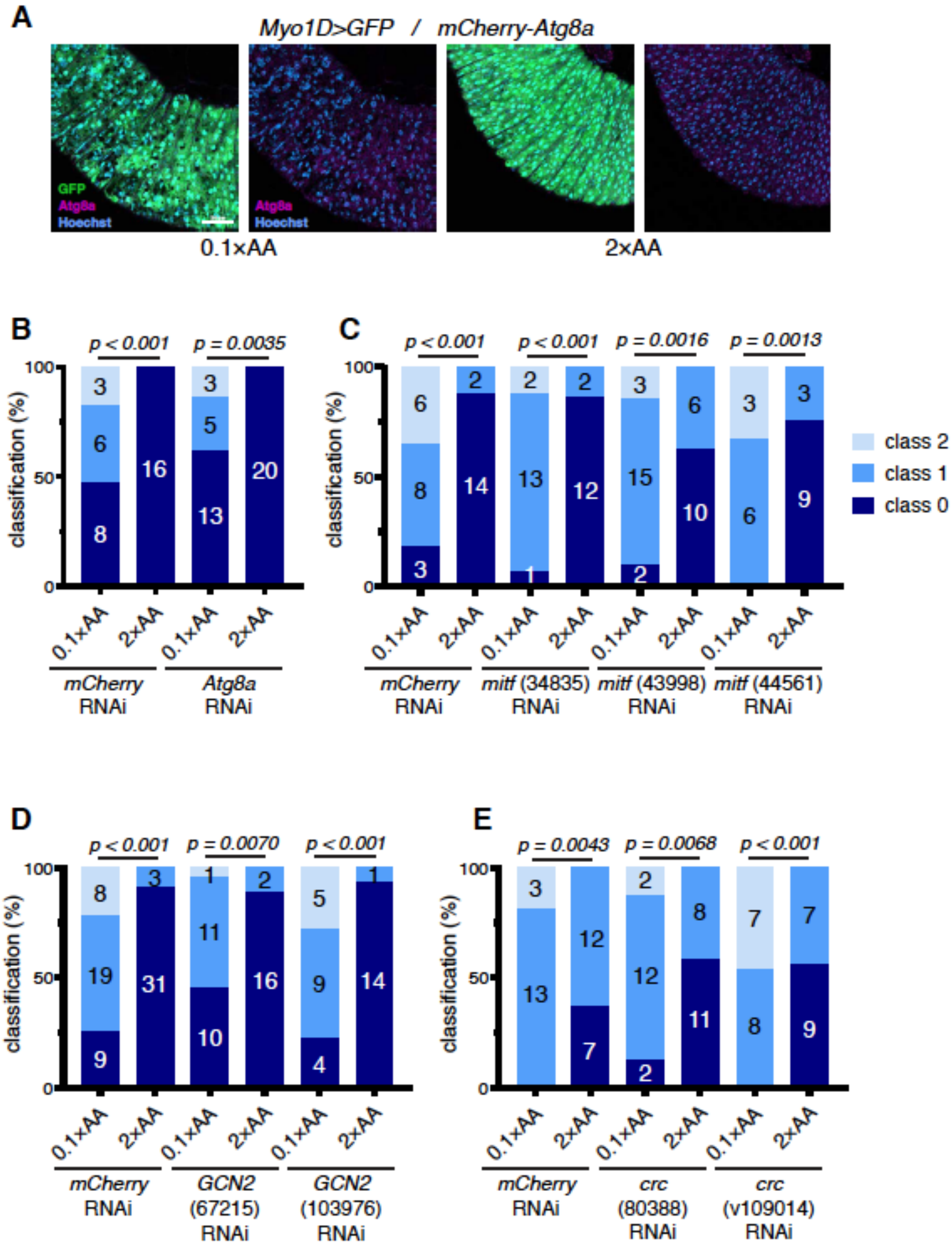

Figure S2 Autophagy or GCN2-ATF4 signaling is not involved in low amino acid-induced erebosis

A. Amino acid concentrations do not alter autophagosome formation detected by mCherry-Atg8a.

B. Inhibition of Atg8a does not affect amino-acid-mediated regulation of erebosis.

C. Inhibition of mtif does not affect amino-acid-mediated regulation of erebosis.

D. Inhibition of GCN2 does not affect amino-acid-mediated regulation of erebosis.

E. Inhibition of crc (ATF4) does not affect amino-acid-mediated regulation of erebosis.

P values were calculated using Fisher’s exact test.

FigS3

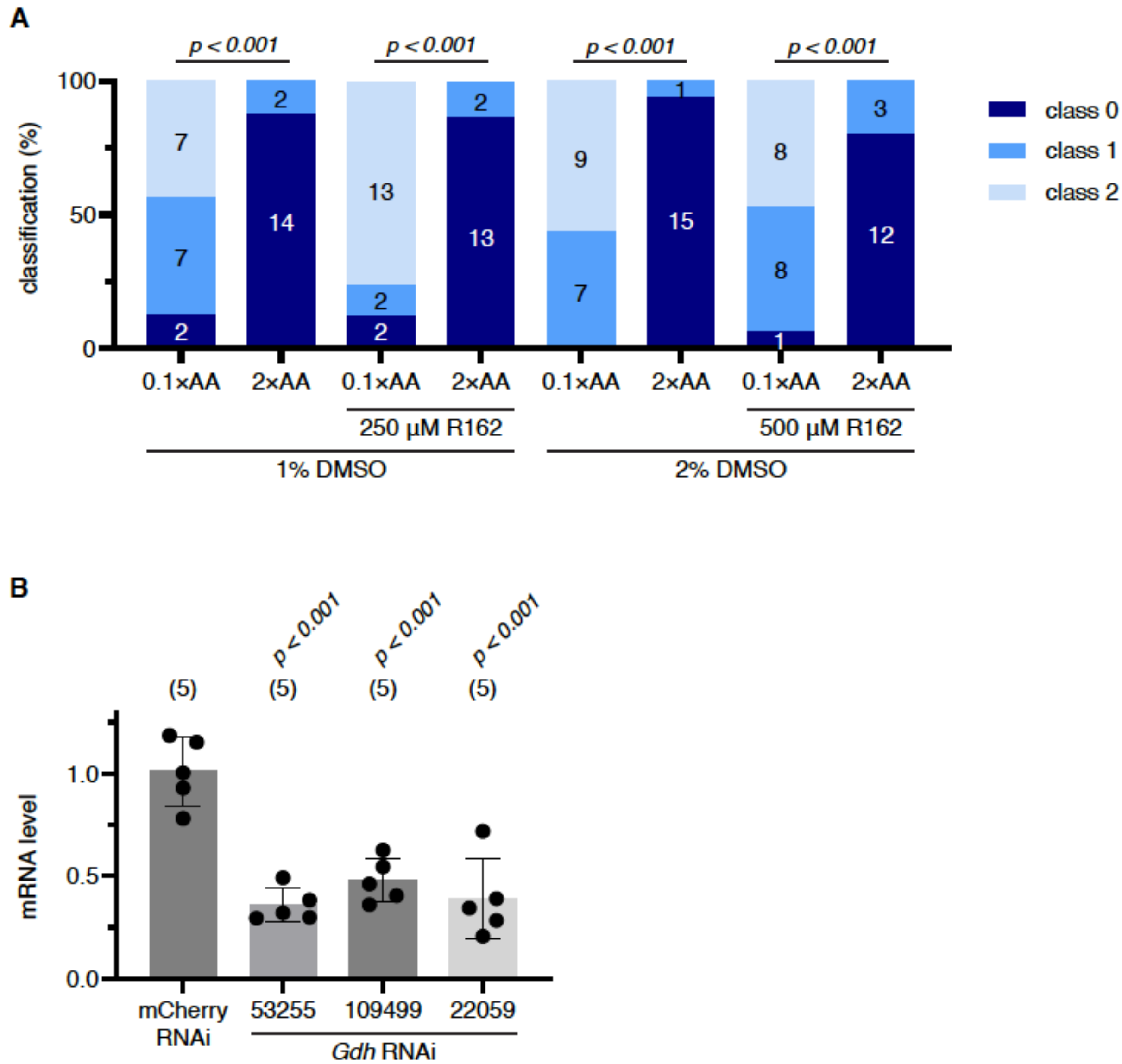

Figure S3 Inhibition of glutamate dehydrogenase, a key enzyme for generating ammonia from glutamate, does not affect amino-acid-mediated regulation of erebosis

A. R162, a GDH inhibitor, does not affect amino-acid–mediated regulation of erebosis.

B. Three independent RNAi lines for Gdh suppress its transcript expression.

P values were calculated using Fisher's exact test (A) and ANOVA with Dunnett's multiple comparison (B).

Fig S4

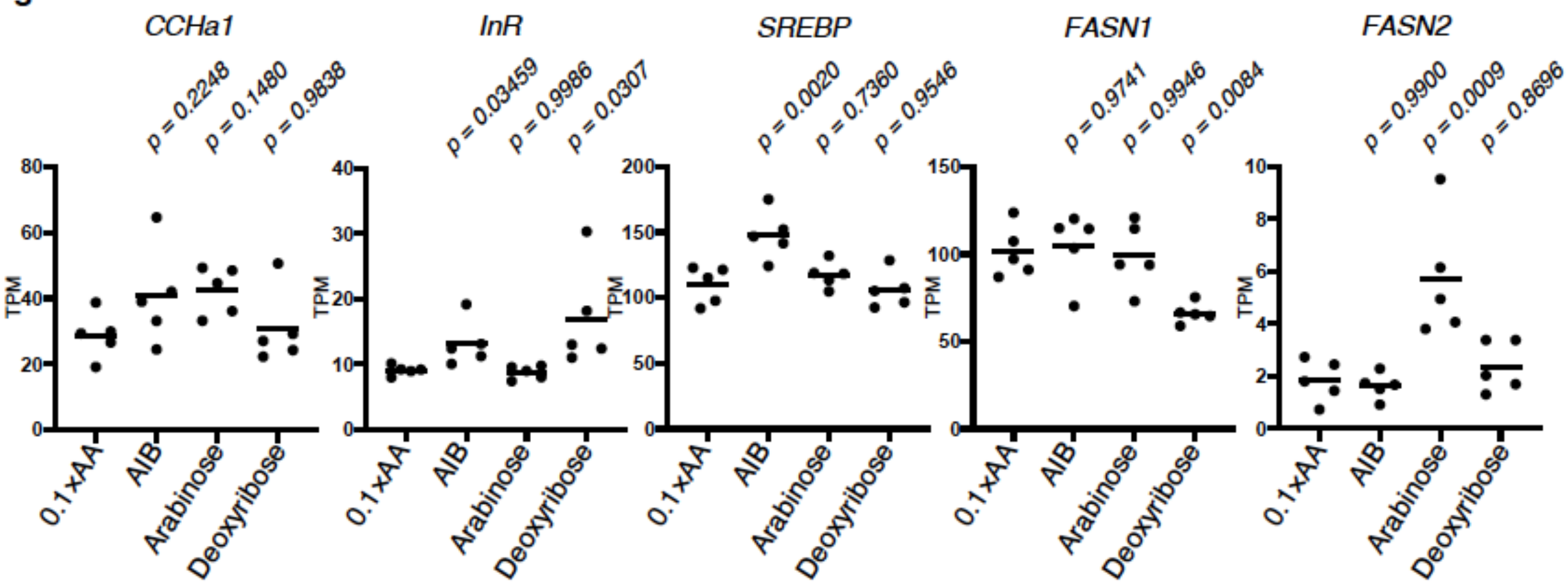

Figure S4 Viscogen molecules do not affect Insulin or Tor signaling consistently
CCHa1 is a high-protein-responsive gene, InR is regulated by Insulin signaling and SREBP/FASN1/FASN2 are regulated by Insulin and Tor signaling. Compared to amino acids (Fig S1B), viscogens have little effects on expression of these genes.
P values were calculated ANOVA with Dunnett's multiple comparison.
